# Clinical Difference between Acute Appendicitis and Acute Right-Sided Colonic Diverticulitis

**DOI:** 10.1155/2020/4947192

**Published:** 2020-09-01

**Authors:** Ji Ho Song, Yong Won Kim, Sanghun Lee, Han Ho Do, Jun Seok Seo, Jeong Hun Lee, Seung Chul Lee

**Affiliations:** ^1^Department of Emergency Medicine, Dongguk University Ilsan Hospital, Dongguk University College of Medicine, Goyang, Republic of Korea; ^2^Department of Emergency Medicine, Kangwon National University College of Medicine, Chuncheon, Republic of Korea

## Abstract

**Background:**

Clinical presentations of acute appendicitis (AA) and acute right-sided colonic diverticulitis (ARCD) are similar. However, the usual treatment for each disease differs between surgical and conservative management. The aim of this study was to identify clinical differences between AA and ARCD.

**Method:**

We performed a single-center retrospective study on adult patients, with uncomplicated AA and ARCD confirmed by computed tomography, who visited an emergency department between March 2018 and August 2019. Clinical variables including past medical history, presented symptoms and signs, and laboratory findings were compared between the two groups. A logistic regression analysis was subsequently performed to differentiate ARCD from AA based on results of univariate analyses.

**Results:**

A total of 212 (79.1%) and 56 (20.9%) patients were enrolled in AA and ARSD groups, respectively. Logistic regression analysis revealed that a past history of diverticulitis [OR: 102.679 (95% CI: 9.964–1058.055), *p* < 0.001] was associated with ARCD, while ketonuria [OR: 2.907 (95% CI: 1.091–7.745), *p*=0.033], anorexia [OR: 21.544 (95% CI: 3.905–118.868), *p* < 0.001], and neutrophilia [OR: 3.406 (95% CI: 1.243–9.336), *p*=0.017] were associated with AA.

**Conclusion:**

Anorexia, neutrophilia, and ketonuria were predictors of AA while a history of diverticulitis was a predictor of ARCD.

## 1. Introduction

Acute appendicitis (AA) and acute right-sided colonic diverticulitis (ARCD) are inflammatory diseases that occur near anatomical locations. Their clinical presentations are similar, making it difficult to differentiate between them [1–3]. The current standard treatment for each disease is different. Appendectomy may be needed for AA while nonoperative conservative treatments such as bowel rest and/or antibiotics are usually used for uncomplicated diverticulitis [4–7]. Nevertheless, ARCD is often misdiagnosed as AA and revealed during surgery [3, 8]. Computer tomography (CT) can be used as a diagnostic imaging tool to avoid unnecessary surgical exploration or for ARCD mimicking AA [9]. However, it has the disadvantages of radiation exposure and cost. Although ultrasound is an alternative image modality to identify these two diseases without radiation hazard, it requires skillful users [10, 11]. Furthermore, episodes of diverticulitis are reported to recur in about 30% of cases. Abdominal image testing for each episode to differentiate between these two diseases can be a burden for the patient [6, 12]. In this respect, only a few studies have verified clinical differences between these two diseases [13, 14]. The purpose of this study was to identify different factors initially presented in the emergency room between AA and ARCD.

## 2. Materials and Methods

### 2.1. Study Design and Population

This single-center retrospective review study was conducted on consecutive Asian patients with age of 18 years old or older who visited the emergency department of a tertiary university hospital between March 2018 and August 2019 in the Republic of Korea, and diagnosis of AA or acute colonic diverticulitis was confirmed by a radiologist through an abdominal CT scan. Exclusion criteria were as follows: (1) patients had complications resulting from appendicitis and diverticulitis verified by CT scan (such as perforation, abscess, and/or fistula), (2) diverticulitis was not included in the right side of the colon, and (3) patients were transferred to another facility.

### 2.2. Study Variables

Clinical data obtained from electronic medical records included age, sex, body mass index (BMI), the time from symptom onset to visiting the emergency room, past medical history of previous diverticulitis or chronic diseases (diabetes, hypertension), gastrointestinal symptoms and signs (right lower quadrant [RLQ] pain, migrated pain to the RLQ, anorexia, nausea and vomiting, diarrhea), physical exams (initial body temperature, RLQ tenderness, rebound tenderness), laboratory findings (complete blood count, serum alanine transaminase [ALT], serum creatinine [Cr], serum C-reactive protein [CRP], urine ketone), Alvarado score and its components, and outcomes (need for hospitalization, length of stay, need for surgical treatment, mortality).

### 2.3. Study Definition

ARCD was defined as diverticulitis originating from primary inflammation of diverticulosis sited at the cecum or ascending colon. A history of diverticulitis was defined if patients were previously diagnosed with any sited colonic diverticulitis before the study period. Anorexia was defined as loss of appetite for food. Elevated ALT was defined as serum ALT over the upper limit of the normal value of 33 IU/L for males or 25 IU/L for females [15]. Elevated Cr was defined as serum Cr over the upper limit of the normal value of 1.29 mg/dL for males or 1.1 mg/dL for females [16]. Elevated CRP was defined as serum CRP concentration over 1.0 mg/dL, indicating significant inflammation [17]. Ketonuria was defined by a positive urine ketone dipstick test. The Alvarado score, a 10-point clinical scoring system, was calculated by adding each score to each clinical factor listed in Table 1 [18].

### 2.4. Statistical Analysis

Study variables of AA and ARCD groups were compared. Continuous variables are presented as median values (interquartile range, IQR). They were compared with the Mann–Whitney test. Nominal data were calculated as percentages based on the frequency of occurrence and compared using Chi-squared or Fisher's exact test as appropriate. Multivariate logistic regression was used to correlate single variables with ARCD. Resulting odds ratios (ORs) and 95% confidence intervals (95% CIs) are presented. A two-sided *p* value of less than 0.05 was considered statistically significant. All statistical analyses were performed using IBM Statistical Package for the Social Sciences (SPSS) software version 24.0 (SPSS, Inc., Chicago, IL, USA).

## 3. Results

During the study period, a total of 231 cases of acute appendicitis and 74 cases of acute colonic diverticulitis cases were diagnosed by abdominal CT scans of adult patients admitted to the emergency department. Among them, complicated cases (12 cases of appendicitis and 3 cases of diverticulitis), cases with non-right-side diverticulitis (*n* = 14), and cases that were transferred out (7 cases of acute appendicitis, 1 case of acute colonic diverticulitis) were excluded. Finally, 212 (79.1%) and 56 (20.9%) patients were enrolled in AA and ARCD groups, respectively.

Patient characteristics including clinical factors associated with each disease group and outcome are shown in Table 2. The AA group had more RLQ pain (94.3% vs. 85.7%, *p*=0.042), ketonuria (40.0% vs. 21.3%, *p*=0.018), anorexia (35.8% vs. 7.1%, *p* < 0.001), RLQ tenderness (98.1% vs. 87.5%, *p*=0.002), rebound tenderness (40.1% vs. 25.0%, *p*=0.043), neutrophilia (62.7% vs. 37.5%, *p* < 0.001), admission care (100% vs. 42.9%, *p* < 0.001), and surgical treatment (99.1% vs. 0%, *p* < 0.001) than the ARCD group. The AA group had higher percentage of neutrophils [78.6 (70.2–84.8) vs. 73.6 (67.1–77.0), *p* < 0.001] and Alvarado scores [6 (4–7) vs. 5 (3–6), *p* < 0.001] than the ARCD group. The ARCD group had longer onset-to-visit intervals [24 (13–48) vs. 11 (3–24) hours, *p* < 0.001], more past histories of diverticulitis (23.2% vs. 0.9%, *p* < 0.001), and higher percentage of lymphocytes [18.2 (13.8–24.9) vs. 14.1(9.3–22.0), *p*=0.003] than the AA group. There was no significant difference in other single clinical variables between the two groups.

Multivariate analysis revealed that factors predictive of ARCD were past history of diverticulitis [OR: 102.679 (95% CI: 9.964–1058.055), *p* < 0.001], anorexia [OR: 0.046 (95% CI: 0.008–0.256), *p* < 0.001], ketonuria [OR: 0.344 (95% CI: 0.129–0.961), *p*=0.033], and neutrophilia [OR: 0.294 (95% CI: 0.107–0.805), *p*=0.017] (Table 3).

## 4. Discussion

Diverticulosis affects approximately 25.1% of Asian population. It accounts for 87.9% of colonic diverticulosis cases involving the right side. This percentage is significantly higher than that in Western countries [19]. ARCD has been reported to occur at a relatively young age. The overall prevalence of diverticulitis is 75% in Asian population [20, 21]. This study was limited to Asians. In the present study, 81.1% of colonic diverticulitis cases were ARCD cases. Our results showed that more surgical treatment was provided for AA, whereas conservative management was more provided for ARCD, consistent with a previous study [7]. Therefore, distinguishing these two diseases will be important for determining a therapeutic plan and avoiding unnecessary surgery for ARCD patients due to misdiagnosis or presumptive diagnosis as AA. The Alvarado scoring system is a classical tool for distinguishing appendicitis from other abdominal diseases [18]. However, even appendicitis can show equivocal Alvarado scores. Some studies have revealed that ARCD patients have higher or broader range of Alvarado scores [22–24]. Therefore, it may not be enough to use this scoring system as a tool to distinguish between these diseases. Although Alvarado scores were different between the two groups in our single variable analysis (median value: 6 points for AA and 5 points for ARCD), it would be difficult to assign clinical meaning because scores of 5 to 6 have an equivocal probability for appendicitis [25]. Currently, few studies have reported clinical differences in symptoms and signs (such as longer symptom duration associated with ARCD and nausea or vomiting, anorexia, migration pain, and RLQ pain associated with AA) between AA and ARCD [13, 14, 22]. Most differences in symptoms might be due to different pathophysiologies and elapsing course of these two diseases. Although both diseases have similar final symptoms due to localized peritonitis, appendicitis has a sequential reaction with prodromal symptoms due to blockage and dilatation of the appendix first. The increase in intraluminal pressure then results in wall necrosis [26]. However, each subjective indicator might have a risk of bias by clinicians or patients. Otherwise, a few studies have reported that some laboratory factors (such as neutrophilia and high CRP) are associated with AA rather than ARCD [13, 14]. Like results of most previous studies, we found that a previous history of diverticulitis was a predictor of ARCD while anorexia and neutrophilia were predictors of AA.

An insufficient number of studies have reported the usefulness of leukocytosis for differentiating between the two diseases and, thus, it remains controversial [22, 27]. Shin et al. [13] have reported an elevated proportion of lymphocytes and a near-normal proportion of segmented neutrophils in ARCD. However, no hypothesis has been suggested to explain this phenomenon. Our results supported the relationship between neutrophilia and AA, and higher fractions of lymphocytes were also related to ARCD. Sasaki et al. [14] have categorized high serum CRP as > 3.0 mg/dL and reported that high serum CRP is associated with ARCD. However, our results did not support such finding. There was no statistical difference in the percentage of high serum CRP (32.5% vs. 37.5%, *p*=0.526) in our study between AA and ARCD groups, even when serum CRP values of over 3.0 mg/dL were used as criteria. Serum CRP is known to peak after 48 hours due to its response to inflammation [28]. Our study and the study by Sasaki et al. [14] differed in the time interval from onset-to-visit in the ARCD patients (24 h vs. 48 h). This could affect blood sample collection time after infection. Therefore, it might be inappropriate to compare these two studies. Moreover, it has been revealed that serum CRP levels will increase over time in appendicitis, raising question as to whether high CRP levels are more relevant to ARCD than to AA if confounding time factors related to CRP increase are not removed [29].

**Figure 1:**
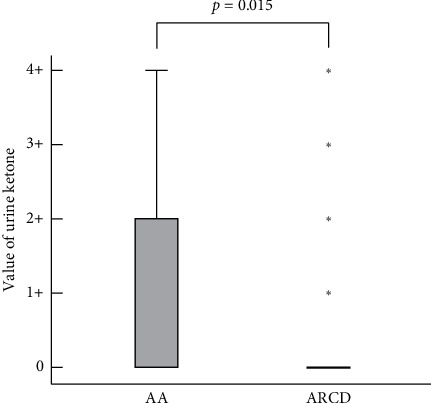
Boxplot showing urine ketone values from dipstick tests for acute appendicitis and acute right-sided colonic diverticulitis patients.

Our results revealed that ketonuria was one of the objective predictors of AA. We presented a boxplot for the distribution of urine ketone values between the two groups as shown in Figure 1. A few studies reported that ketonuria was seen in 12% to 45% of the AA cases, but there have been no comparative studies between AA and ARCD [30, 31]. Ketones are known as end-products of fatty acid metabolism. Ketonuria indicates that the body is excessively using fat over carbohydrates as the major source of energy [32]. Ketonuria could arise from dietary conditions (such as fasting, nausea and vomiting, and anorexia) and metabolic conditions (including type-1 diabetes, fever, and pregnancy) [33]. In our study, there were no patients with type-1 diabetes or pregnant women. There was no significant difference in body temperature between the two groups either. Therefore, the probable cause of ketonuria in appendicitis might be related to dietary conditions such as anorexia because these conditions were found more in the AA group than in the ARCD group. When we made a simple rule for predicting ARCD that included objective clinical factors such as no ketonuria and no neutrophilia with the presence of a history of diverticulitis, the calculated positive predictive value (PPV) was 87.5% (95% CI: 46.7–98.2%), the negative predictive value (NPV) was 95.2% (95% CI: 94.6–95.7%), and the specificity was 99.8% (95% CI: 99.3–99.9%). This rule might be a useful tool for distinguishing ARCD from AA.

This study has some limitations. First, this study was conducted on an Asian population at a single center in the Republic of Korea. Therefore, results of this study might not represent all races and nations. However, the presentation of diverticulitis in the Asian population is unusual because it most commonly involves the right side of the colon. The exact pathological mechanism of diverticular disease is unclear, although several theories related to genetics, diet, motility, and microbiome that might be affected by individual races and cultures have been presented [34]. Therefore, this study has some clinical implications because not many studies have been reported in an Asian population.

Second, because this study was a retrospective study rather than a confirmative study, data or cases might be missing. Specifically, for subjective symptoms and signs, it was difficult to accurately describe the intensity or presence without a proper prospective protocol.

Third, ketonuria is roughly correlated with serum ketone concentrations, but the absence of ketonuria does not mean the absence of blood ketone bodies. However, we could not obtain the serum ketone concentrations to distinguish between AA and ARCD as that analysis was not included in the routine ER blood tests because it is more expensive than urine analysis and has not been proven useful in the diagnosis of these diseases. Further studies that quantitatively analyze these factors are needed.

## 5. Conclusion

Our results suggest that anorexia, neutrophilia, and ketonuria are useful predictors of AA, but not ARCD. Conversely, a history of diverticulitis was a useful predictor of ARCD, but not AA. If a history of diverticulitis is present without neutrophilia or ketonuria, then the PPV is 87.5% and the specificity is 99.8% for ARCD. Our findings could be used for differential diagnosis between AA and ARCD to reduce unnecessary additional imaging studies for ARCD.

## Figures and Tables

**Table 1:** Alvarado score.

Components	Score
Anorexia	1
Nausea or vomiting	1
Migrating pain to RLQ	1
RLQ tenderness	1
Rebound tenderness	2
Elevated temperature (>37.3°)	1
Leukocytosis >10,000/mm^3^	2
Neutrophilia >75%	1
Total	10

RLQ: right lower quadrant.

**Table 2:** General characteristics.

Parameters		Total *N* = 268	AA group *N* = 212 (79.1%)	ARCD group *N* = 56 (20.9%)	*p* value
Age (yr)		40 (31–52)^*∗*^	39 (30–53)^*∗*^	41 (33–47)^*∗*^	0.224
Male gender, no. (%)		131 (48.9)	107 (50.5)^*∗*^	24 (42.9)^*∗*^	0.368
BMI (kg/m^2^)		23.3 (20.7–25.9)^*∗*^	23.4 (20.9–25.9)^*∗*^	22.4 (20.5–25.9)^*∗*^	0.389
Onset-to-visit interval (hr)		15 (4–47)^*∗*^	11 (3–24)^*∗*^	24 (13–48)^*∗*^	<0.001

Past medical history					
Diabetes, no. (%)		11 (4.1)	8 (3.8)	3 (5.4)	0.704
Hypertension, no. (%)		18 (6.7)	12 (5.7)	6 (10.7)	0.226
History of diverticulitis, no. (%)		15 (5.6)	2 (0.9)	13 (23.2)	<0.001

Symptoms and signs					
Body temperature(°C)		36.8 (36.5–37.3)^*∗*^	36.8 (36.5–37.3)^*∗*^	36.8 (36.5–37.1)^*∗*^	0.432
RLQ pain, no. (%)		248 (92.5)	200 (94.3)	48 (85.7)	0.042
Diarrhea, no. (%)		48 (17.9)	34 (16.0)	14 (25.0)	0.169
Constipation, no. (%)		8 (3.0)	4 (1.9)	4 (7.1)	0.062

Laboratory findings					
WBC		11,470 (9,110–14,270)^*∗*^	11,740 (9,155–14,550)^*∗*^	11,160 (8,920–12,900)^*∗*^	0.224
Absolute neutrophil count		8,860 (6,340–11,670)^*∗*^	9,210 (6,410–11,830)^*∗*^	7,960 (6,130–9,930)^*∗*^	0.063
Neutrophil (%)		77.5 (69.1–83.3)^*∗*^	78.6 (70.2–84.8)^*∗*^	73.6 (67.1–77.0)^*∗*^	<0.001
Lymphocyte (%)		14.8 (10.5–22.9)^*∗*^	14.1 (9.3–22.0)^*∗*^	18.2 (13.8–24.9)^*∗*^	0.003
Hb (g/dL)		13.9 (12.7–14.9)^*∗*^	14.0 (12.8–15.0)^*∗*^	13.8 (12.3–14.7)^*∗*^	0.374
Hct (%)		40.8 (37.5–43.9)^*∗*^	40.8 (37.7–43.9)^*∗*^	40.7 (36.9–43.8)^*∗*^	0.920
Elevated ALT, no. (%)		36 (13.4)	31 (14.6)	5 (8.9)	0.286
Elevated Cr, no. (%)		6 (2.2)	5 (2.4)	1 (1.8)	1.000
Elevated CRP, no. (%)		155 (57.1)	123 (58.0)	32 (57.1)	1.000
CRP (mg/dL)		1.5 (0.4–4.6)^*∗*^	1.4 (0.3–4.2)^*∗*^	2.2 (0.8–4.6)^*∗*^	0.138
Ketonuria, no. (%)		90/247 (36.4)	80/200 (40.0)	10/47 (21.3)	0.018

Alvarado score and components					
Alvarado score		6 (4–7)^*∗*^	6 (4–7)	5 (3–6)^*∗*^	<0.001
Migration pain, no. (%)		49 (18.3)	44 (20.8)	5 (8.9)	0.051
Anorexia, no. (%)		90 (33.6)	76 (35.8)	4 (7.1)	<0.001
Nausea or vomiting, no. (%)		92 (32.7)	78 (35.1)	14 (23.7)	0.153
RLQ tenderness, no. (%)		257 (95.9)	208 (98.1)	49 (87.5)	0.002
Rebound tenderness, no. (%)		99 (36.9)	85 (40.1)	14 (25.0)	0.043
Body temperature ≥37.3°C, no. (%)		71 (26.5)	59 (27.8)	12 (21.4)	0.396
Leukocytosis, no. (%)		179 (66.8)	142 (67.0)	37 (66.1)	1.000
Neutrophilia, no. (%)		154 (57.5)	133 (62.7)	21 (37.5)	0.001

Outcomes					
Admission care, no. (%)		236 (88.1)	212 (100)	24 (42.9)	<0.001
Hospital days		5 (4–6)^*∗*^	5 (4–6)^*∗*^	5 (4–6)^*∗*^	0.574
Surgical treatment, no. (%)		211 (78.7)	210 (99.1)	0	<0.001
Mortality, no. (%)		1 (0.4)	1 (0.5)	0	1.000

^*∗*^Median (interquartile range); AA: acute appendicitis; ARCD: acute right-sided colonic diverticulitis; BMI: body mass index; RLQ: right lower quadrant; WBC: white blood cell; Hb: hemoglobin; Hct: hematocrit; LDH: lactate dehydrogenase; ALT: alanine transaminase; Cr: creatinine; CRP: C-reactive protein.

**Table 3:** Multivariate analysis of predictors of ARCD compared to AA.

Predictors of acute ARCD	Odds ratio	95% CI	*p* value
Onset-to-visit interval (hr)	1.000	0.999–1.001	0.800
History of diverticulitis	102.679	9.964–1058.055	<0.001
Anorexia	0.046	0.008–0.256	<0.001
RLQ pain with tenderness	0.311	0.094–1.029	0.056
Rebound tenderness	0.455	0.182–1.138	0.092
Ketonuria	0.344	0.129–0.916	0.033
Neutrophilia	0.294	0.107–0.805	0.017

CI: confidence interval; ARCD: acute right-sided colonic diverticulitis; AA: acute appendicitis; RLQ: right lower quadrant.

## Data Availability

All datasets used and/or analyzed in the current study are available from the corresponding author upon reasonable request.
